# A web-based pilot randomized controlled trial to test the efficacy of education and contact-based interventions in reducing public suicide stigma

**DOI:** 10.1186/s12888-024-06406-7

**Published:** 2025-01-23

**Authors:** Nathalie Oexle, Matthias Lühr, Daniele Valacchi, Nicolas Rüsch

**Affiliations:** Department of Psychiatry II, University of Ulm and BKH Günzburg, Parkstrasse 11, 89073 Ulm, Germany

**Keywords:** Suicide prevention, Stigma, Normalization, Attitudes, RCT

## Abstract

**Background:**

Interventions to reduce public suicide stigma (i.e. negative attitudes towards persons affected by suicide/suicidality) could contribute to suicide prevention. However, such interventions could unintentionally increase suicide normalization (i.e. liberal attitudes towards suicide) and therefore increase suicide risk. We aimed to test the efficacy of education- and contact-based interventions delivered online via video or text on both public suicide stigma and suicide normalization.

**Methods:**

We conducted a web-based randomized controlled trial among *N* = 2,043 participants recruited from an established online research panel. Participants were randomized into six groups, receiving either one of four contact- or education-based interventions that were transferred via text or video (contact text, contact video, education text, education video) or control group content (contact control, education control). Information about suicide stigma and suicide normalization were collected directly before (t_0_) and after intervention participation (t_1_) as well as about two weeks later (t_2_). To explore the attractiveness of the provided intervention material, we used Brown-Mood’s median test to compare the times participants spent with the provided intervention material in each group. We then used linear mixed models to compare effects on suicide stigma and suicide normalization between intervention groups and control groups.

**Results:**

Median times spent with provided material were generally longer among participants exposed to video material than among participants exposed to text material, and among participants in contact-based interventions than among participants in education-based interventions. We did not observe stronger effects in intervention groups compared to control groups on suicide stigma or suicide normalization. Surprisingly, suicide stigma and suicide normalization appeared to decrease from t_0_ to t_1_ in both intervention and control groups.

**Conclusion:**

Our findings suggest a higher attractiveness of video- and contact-based material compared to text- and education-based material. However, none of the interventions had a significant effect on public suicide stigma or suicide normalization. Future research should explore innovative and safe approaches to reduce public suicide stigma. Experimental studies may focus on interventions with higher attractiveness (i.e. video and contact-based interventions), use interventions with higher intensity (i.e. longer interventions, more repetitions), and assess suicide stigma with implicit measures to avoid response bias.

**Trial registration:**

The RCT was registered at clinicaltrials.gov on February 11th, 2021 (NCT04756219).

**Supplementary Information:**

The online version contains supplementary material available at 10.1186/s12888-024-06406-7.

## Introduction

Worldwide, about 700,000 people die by suicide each year and while national suicide rates tend to be relatively stable over time, they do vary across cultures. For example, in 2019 the age-standardized suicide rate for Europe was 10.5 per 100,000, compared to 6.4 per 100,000 in the Eastern Mediterranean region [1]. While there surely are various reasons for such heterogeneity, emerging evidence, such as the Cultural Theory and Model for Suicide, suggests that cultural values about and social attitudes towards suicide can contribute to observed variations in national suicide rates [2, 3]. In line with that, scholars previously argued that successful suicide prevention requires a public health approach, i.e. efforts to target relevant factors (including societal attitudes towards suicide) among members of the general population [4]. Existing research particularly highlighted the relevance of targeting public suicide stigma (i.e. negative attitudes towards persons affected by suicide/suicidal thoughts and behavior) for suicide prevention [5].

In general, stigmatization is a process during which minority groups are systematically devalued and discriminated based on certain personal attributes such as their skin color, mental health status or experiences with suicidality (i.e. suicidal thoughts and/or behavior) [6]. This process entails three steps, starting with stereotypes that are associated with the beforementioned personal attributes (e.g. “persons who attempt suicide just want attention”). If agreed to, stereotypes can lead to negative emotional reactions, i.e. prejudice (e.g. “yes, persons who attempt suicide just want attention and are therefore annoying”), as well as discrimination (e.g. social avoidance) [7]. Stigma can be present among members of the general public (i.e. public stigma) as well as manifest as self-stigma (i.e. self-devaluation) among stigmatized individuals [7]. For majority members (i.e. those who are not members of the stigmatized social group), stigma can have various beneficial functions, including exploitation and domination (‘keep people down’), avoidance of disease (‘keep people away’) or the enforcement of social norms (‘keep people in’) [7, 8]. In the western world, suicide was judged negatively since late antiquity and remains a taboo and stigmatized topic until today, with detrimental effects for suicide prevention. For example, public suicide stigma was found to hinder the implementation of programs to prevent suicide [9] as well as to reduce help-seeking for suicidality [10, 11]. Additionally, emerging evidence suggests that the perception of public suicide stigma can increase emotional distress and suicidality among persons who survived a suicide attempt [12] and those who lost a loved one to suicide [13], who are both at increased risk to die by suicide [14]. Such evidence indicates that efforts to reduce public suicide stigma among members of the general population could contribute to suicide prevention.

However, as stated above, stigmatization can have beneficial functions for majority members, including the enforcement of social norms (e.g. suicidal behavior is not acceptable). Interventions to reduce public suicide stigma are therefore at risk to unintentionally increase suicide normalization (i.e. liberal attitudes towards suicide) among members of the general population. This could limit the beneficial effect of such interventions for suicide prevention, as several cross-national investigations found that approving or liberal attitudes about suicide were associated with increased national suicide rates [15, 16]. In line with this hypothesis, we recently observed an inverse link between public suicide stigma and suicide normalization among members of the general population [17]; however, analyzed data were cross-sectional and forbid any causal interpretation. To date, studies investigating whether interventions to reduce public suicide stigma also lead to changes in suicide normalization are not available.

Research from related fields suggests two approaches to reduce public suicide stigma, both of which can be presented via text or video [18, 19]. Education-based material aims to reduce public suicide stigma by disconfirming stereotypes and busting the myths surrounding suicide (i.e. increase suicide-related knowledge), and contact-based material targets prejudice by establishing interpersonal contact with members of the stigmatized group. Additionally, despite great relevance for suicide prevention, little is known about the effectiveness of these approaches in reducing public suicide stigma as well as their impact on additional relevant outcomes such as suicide normalization. Aspects of education- and contact-based approaches have however been considered in some studies investigating the beneficial or harmful effects of news media articles discussing suicide. Williams and colleagues investigated the effects of a suicide news article about a person who died by suicide including psychoeducational information and observed no effects on attitudes about suicide, attitudes to seek help for suicidality or suicide-related knowledge [20]. Till and colleagues [21] conducted a web-based randomized controlled trial (RCT) testing the effects of educative suicide news articles and found them to be safe to use (i.e. no increase in suicide risk); however, they observed no effects on stigmatizing attitudes about suicide or help-seeking intentions. In line with previous research, the effectiveness of such interventions is likely influenced by the extent to which provided materials attract the interest and attention of the target population, for example due to personal relevance, aversion or approval [22]. While such intervention attractiveness can be measured in different ways (e.g. qualitative feedback, recall tests, facial expression analysis), one rough indicator that has been used in previous research is the time spent with provided material [23].

In summary, despite relevance for suicide prevention, the impact of education or contact-based interventions on public suicide stigma and suicide normalization remains unclear. While most existing studies used text-based material, effects could differ for video-based interventions, which might increase interest and attention among study participants. To contribute to the existing literature, the presented study aimed to investigate the effect of contact- and education-based interventions transferred via text or video on public suicide stigma and suicide normalization among members of the general population, as well as explore the attractiveness of these interventions.

## Methods

### Procedure and participants

Methods and findings are reported according to the Consolidated Standards of Reporting Trials (CONSORT) guidelines [24]. This paper is based on data derived from the RISE study (RISE: *Reducing* public *suicide* stigma). We conducted a single-blind multi-arm online RCT to test the efficacy of contact and education-based materials to reduce public suicide stigma. Participants were recruited from a German online research panel (Respondi mingle) and quotas were successfully applied, so that the studied sample reflects the composition of the German general population regarding age, gender, education and region. All data collection (incl. randomization & interventions) took place on the online research platform SoSci Survey (www.soscisurvey.de) between February and March 2021. Participants had to be a least 18 years old and indicate low suicide risk based on the following item adapted from the Patient Health Questionnaire-9 (PHQ-9) [25]: “During the last three months, did you oftentimes think that you would be better off dead?”. Persons who answered with “yes” were excluded, received digital information about mental health services and were offered to talk to a psychiatrist (NR). A manuscript presenting baseline data was recently published [17].

**Fig. 1:**
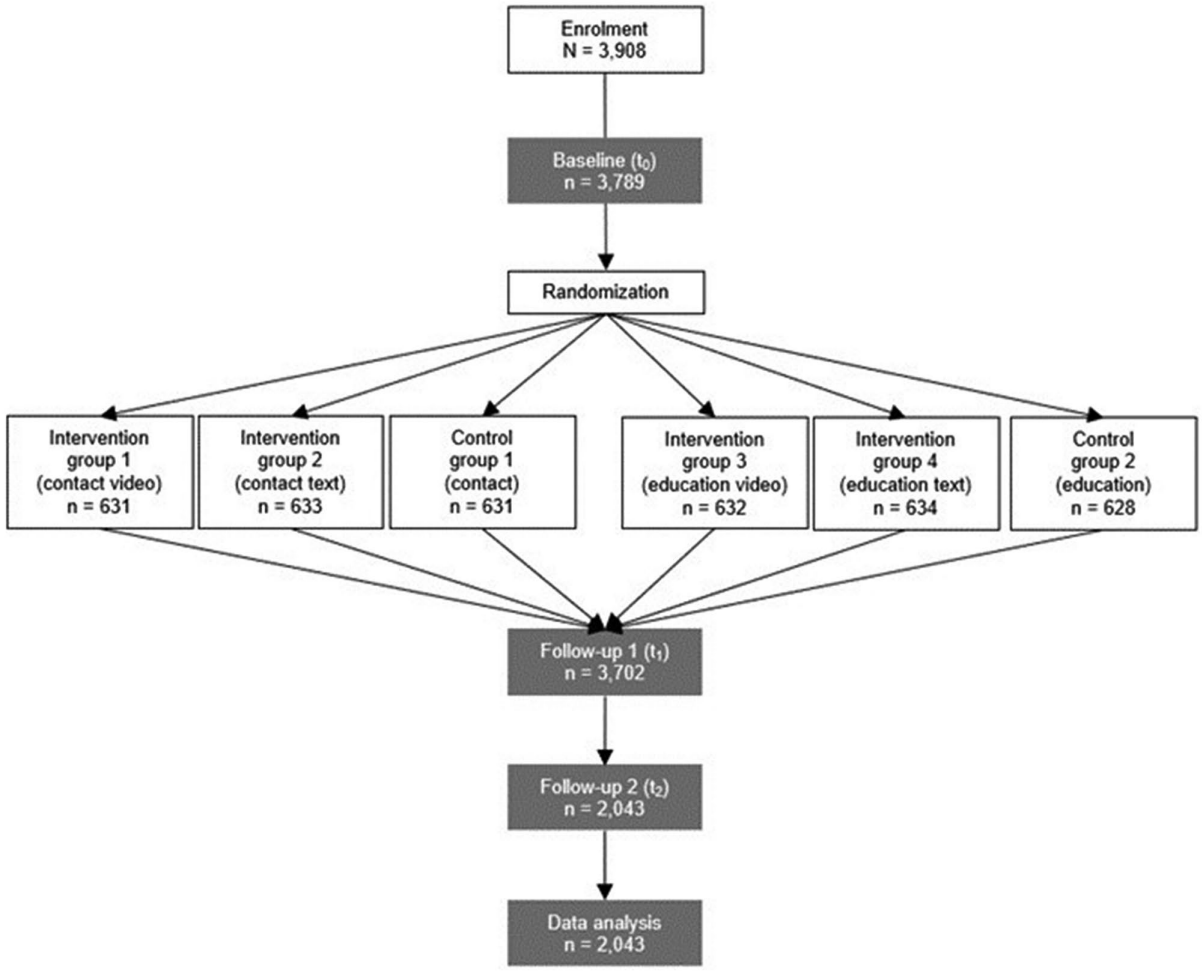
Study flow chart

Persons registered on Respondi mingle were invited to participate in a research study and redirected to an online survey hosted by the research platform SoSci Survey. Interested persons received digital study information including the topic of the study (societal attitudes about suicide) and were asked to provide online informed consent. In total, 3,789 persons met the inclusion criteria. After completing the baseline data collection (t_0_), participants were randomized (urn randomization with replacement, automatic feature within SoSci Survey) to receive one of four interventions (contact video, contact text, education video, education text) or control group content (contact control, education control). Participants and researchers were masked to group assignment. Directly after the intervention/control condition, post-intervention data collection (t_1_) took place. Based on an anonymous code system used by Respondi mingle, participants were re-invited to participate in a follow-up data collection (t_2_) about two weeks after t_0_/t_1_ (see Fig. 1 for a study flow chart). Small reimbursements were paid by Respondi mingle based on participants’ individual response times.

### Sample size

We conducted an *a prori* power analysis using an online calculator [26] for a mixed ANOVA model with three repeated measures and six groups. Based on alpha = 0.05 and beta = 0.80, *N* = 396 persons would be required to detect small to medium effects (ƞ^2^ = 0.04). A sample size estimation for linear mixed models with similar specifications using GLIMMPSE 3.1.2 [27] yielded a required sample size of *N* = 327 participants.

### Interventions

Following existing guidelines for contact-based anti-stigma programs [28], we developed two contact-based interventions (video vs. text) to enable interpersonal contact with a suicide attempt survivor in order to disconfirm stereotypes and reduce prejudice about this group. For the video-based intervention (CV), participants watched a short video (approx. 6 min) of a male suicide attempt survivor discussing his experiences with suicidality, road to recovery and experiences with stigma. For the text-based intervention (CT), participants read a short story (826 words) with similar content. Participants in the control group (CC) read a short story (657 words) of a man who survived a heart attack discussing his experiences and road to recovery.

Similar to the contact-based interventions, we developed two education-based interventions using text or video. Their general aim was to educate participants about suicide to disconfirm stereotypes about individuals who experience suicidality. For the video intervention (EV; approx. 6 min), participants watched a psychiatrist (NR) presenting facts about suicide statistics, suicide warning signs and risk factors, as well as about what people can do when being confronted with own or others’ suicidal thoughts. The text intervention (ET; 657 words) provided similar content, but in written text. Participants in the control group read a text (EC; 588 words) that included information about cardiac arrest (i.e. definition, prevalence, risk factors, symptomatology).

We did not control the time participants spent with their allocated content. Instead, participants were allowed to continue with the remaining questionnaires whenever they felt ready, which resulted in heterogenous intervention times (for an overview, see Table 1), but allowed us to investigate intervention attractiveness.

**Table 1:** Differences in intervention times among intervention groups and results of Brown-Mood’s median test, *N* = 2,043

Difference test	Expected intervention time in seconds	Median intervention time in seconds (MAD)	Median intervention exposure score (MAD)	z	df	*p*
Contact Control vs.	154.5	162.0 (91.92)	0.05 (0.59)	3.46	1	<0.001
Contact Text	216.5	188.0 (136.40)	−0.13 (0.63)			
Contact Control vs.	154.5	162.0 (91.92)	0.05 (0.59)	0.15	1	0.879
Contact Video	370.0	386.0 (32.62)	0.04 (0.09)			
Contact Text vs.	216.5	188.0 (136.40)	−0.13 (0.63)	−5.21	1	<0.001
Contact Video	370.0	386.0 (32.62)	0.04 (0.09)			
Education Control vs.	170.5	79.0 (77.10)	−0.54 (0.45)	−0.39	1	0.698
Education Text	195.0	92.0 (94.89)	−0.53 (0.49)			
Education Control vs.	170.5	79.0 (77.10)	−0.54 (0.45)	−17.29	1	< 0.001
Education Video	344.0	361.0 (16.31)	0.05 (0.05)			
Education Text vs.	195.0	92.0 (94.89)	−0.53 (0.49)	−15.34	1	< 0.001
Education Video	344.0	361.0 (16.31)	0.05 (0.05)			
Contact Control vs.	154.5	162.0 (91.92)	0.05 (0.59)	10.14	1	< 0.001
Education Control	170.5	79.0 (77.10)	−0.54 (0.45)			
Contact Text vs.	216.5	188.0 (136.40)	−0.13 (0.63)	6.23	1	<0.001
Education Text	195.0	92.0 (94.89)	−0.53 (0.49)			
Contact Video vs.	370.0	386.0 (32.62)	0.04 (0.09)	−0.64	1	0.521
Education Video	344.0	361.0 (16.31)	0.05 (0.05)			

MAD  =  Median Absolute Deviation

### Measures

Suicide stigma (i.e. negative attitudes towards people who experienced suicidality) was assessed by the 8-item stigma subscale of the German short version of the Stigma of Suicide Scale [29]. Participants rated their agreement with eight stereotypes about persons who attempt suicide (e.g. immoral) from 1 (strongly disagree) to 5 (strongly agree). A total suicide stigma mean score was calculated with higher scores indicating more negative attitudes (Cronbach’s α at baseline = 0.88).

Suicide normalization (i.e. liberal attitudes towards suicide) was assessed by the 8-item Right To Commit Suicide subscale of the Cognitions Concerning Suicide Scale [30]. Participants rated their agreement with eight statements (e.g. “When life consists of intolerable pain, suicide is an acceptable alternative.”) from 0 (I disagree) to 5 (I agree). Items were reverse coded when necessary and a total normalization of suicide mean score was calculated with higher scores indicating increased normalization of suicide (Cronbach’s α at baseline = 0.75).

We also measured participants’ age in years, gender (male/female/diverse), education level (low: non or vocational education, middle: high school degree, high: university degree), lifetime suicidality (yes/no; defined as having experienced suicidal thoughts or survived a suicide attempt at least once in one’s life) and current suicidal thoughts (measured by one item adapted from the PHQ-9 related to suicidal thoughts in the past 2 weeks [25]).

### Data analysis

To investigate the attractiveness of contact- and education-based text and video interventions, we compared the time participants spent with provided intervention materials across groups. Aiming to ensure the comparability of these times, we calculated an intervention exposure score for each participant as follows: (1) We calculated the deviation between expected intervention times and personal intervention times in seconds. Expected intervention times were either defined as the length of the respective video (for video interventions), or the respective median reading time of eight research assistants (for text interventions). (2) To adjust for differences in expected intervention times across interventions, we divided deviation scores by the expected intervention time. Hence, the resulting intervention exposure score has a value of 0 when it concurs with the expected intervention time. In a next step, we compared medians of the intervention exposure scores across intervention groups using Brown-Mood’s median test [31] and Bonferroni corrected p-values. A comparison of means was not feasible due to extreme outliers in some intervention groups. We compared medians of the intervention exposure scores within contact-based groups (CC vs. CV, CC vs. CT, CV vs. CT), within education-based groups (EC vs. EV, EC vs. ET, EV vs. ET), and within each condition (CC vs. EC, CT vs. ET, CV vs. EV).

We used linear mixed modeling to test the efficacy of contact- and education-based text and video interventions in reducing suicide stigma, as well as their impact on suicide normalization. We set up separate models for type of intervention (contact-based, education-based) and for comparisons between time points (t_0_ vs. t_1_, t_0_ vs. t_2_), which resulted in 4 models for each outcome (i.e. 8 models in total). We introduced dummy coded variables for text intervention (0 = no text intervention, 1 = text intervention) and for video intervention (0 = no video intervention, 1 = video intervention) into the models and used the control content group as the reference group. Interactions between Time and these dummy coded variables were included to test whether changes in suicide stigma and suicide normalization from baseline to post-intervention (t_0_ vs. t_1_) and from baseline to follow-up (t_0_ vs. t_2_) differ between the intervention groups and the control group. Compared to traditional analysis of variance (ANOVA), linear mixed models represent an alternative method of analysis of repeated measures that requires less assumptions to be met, is easier to interpret, and accounts for within-person correlations of repeated measures [32]. All analyses were conducted using R version 4.1.3. as well as R package lme4 (v1.1.35.3) to run linear mixed models [33].

## Results

In total, 4,418 individuals were assessed for eligibility. We excluded 510 persons who did not meet inclusion criteria (younger than 18 years old: *n* = 9; increased levels of suicidal thoughts during the past three months: *n* = 501) and another 119 individuals who dropped out before randomization, leaving 3,789 participants for randomization (CV: *n* = 631, CT: *n* = 633, CC: *n* = 631, EV: *n* = 632, ET: *n* = 634, EC: *n* = 628). Another 87 individuals dropped out after randomization, leaving 3,702 persons who completed t_0_ and t_1_ data collections and who were invited about two weeks later to participate in t_2_. A total of 1,436 persons did not respond to this invitation (*n* = 1,283), did not provide consent (*n* = 135) or dropped out (*n* = 223), leaving 2,043 persons for data analyses. Table 2 shows descriptive statistics of sociodemographic and outcome measures by allocated group. The six groups did not differ significantly in age, current suicidal thoughts, lifetime suicidality, level of education, suicide stigma at t_0_, and suicide normalization at t_0_. However, the CC and ET groups included significantly more women than the other groups.

**Table 2:** Descriptive statistics by intervention group, *N* = 2,043

Baseline variables	Contact Control *N* = 355	Contact Text *N* = 321	Contact Video *N* = 345	Education Control *N* = 338	Education Text *N* = 314	Education Video *N* = 370	χ^2^/F^a^
Age, *M* (*SD*)	49.68 (15.89)	50.17 (14.70)	48.50 (15.31)	50.22 (15.49)	49.47 (15.42)	48.35 (16.09)	0.47^b^
Gender, *n* (%)							27.41^c^
Female Male Diverse	189 (53.2%) 166 (46.8%) 0 (0.0%)	142 (44.2%) 175 (54.5%) 4 (1.2%)	159 (46.1%) 186 (53.9%) 0 (0.0%)	158 (46.7%) 180 (53.3%) 0 (0.0%)	172 (54.8%) 142 (45.2%) 0 (0.0%)	184 (49.7%) 185 (50.0%) 1 (0.3%)	
Current Suicidal Thoughts, *n* (%)							4.71^b^
No Yes	289 (81.4%) 66 (18.6%)	270 (84.1%) 51 (15.9%)	270 (78.3%) 75 (21.7%)	281 (83.1%) 57 (16.9%)	257 (81.8%) 57 (18.2%)	298 (80.5%) 72 (19.5%)	
Lifetime Suicidality, *n* (%)							9.75^b^
No Yes	241 (67.9%) 114 (32.1%)	246 (76.6%) 75 (23.4%)	231 (67.0%) 114 (33.0%)	245 (72.5%) 93 (27.5%)	222 (70.7%) 92 (29.3%)	259 (70.0%) 111 (30.0%)	
Education, *n* (%)							16.10^b^
Low Middle High	144 (40.6%) 107 (30.1%) 104 (29.3%)	133 (41.4%) 96 (29.9%) 92 (28.7%)	113 (32.8%) 123 (35.7%) 305 (31.6%)	140 (41.4%) 97 (28.7%) 101 (29.9%)	101 (32.2%) 112 (35.7%) 101 (32.2%)	131 (35.4%) 112 (30.3%) 127 (34.3%)	
Suicide stigma t_0_, *M* (*SD*)	2.00 (0.80)	2.03 (0.87)	2.06 (0.82)	1.97 (0.74)	1.93 (0.78)	1.94 (0.81)	1.57^b^
Suicide normalization t_0,_*M* (*SD*)	2.11 (1.05)	2.12 (0.98)	2.15 (1.02)	2.09 (0.98)	2.08 (1.02)	2.16 (1.02)	0.21^b^

^a^Values represent χ^2^-values calculated from χ^2^-tests or *F*-values calculated from variance analysis testing differences among intervention groups

^b^*p* > 0.050

^c^*p* = 0.002

There were no differences between completers (*n* = 2,043) and those who dropped out after randomization (*n* = 1,746) in suicide normalization at t_0_ (t(3,771.9) = -0.08, *p* = .935), gender (χ^2^ = 0.07, df = 2, *p* = .964), lifetime suicidality (χ^2^ = 1.02, df = 1, *p* = .312), and contact group assignment (χ^2^ = 4.15, df = 2, *p* = .125). However, we observed differences between these two groups regarding age (t(3719.7) = 15.34, *p* < .001), suicide stigma at t_0_ (t(3628.6) = -6.25, *p* < .001), current suicidal thoughts (χ^2^ = 15.79, df = 1, *p* < .001), education (χ^2^ = 31.25, df = 2, *p* < .001), and education group assignment (χ^2^ = 10.37, df = 2, *p* = .006). Specifically, individuals who dropped out were younger (*M* = 41.71, *SD* = 15.16 vs. *M* = 49.37, *SD* = 15.51), reported higher suicide stigma (*M* = 2.16, *SD* = 0.85 vs. *M* = 1.99, *SD* = 0.80), were more likely to report current suicidal thoughts (23.8% vs. 18.5%), had a higher education (37.5% vs. 31.0%), and were less likely assigned to the education video intervention (30.0% vs. 36.2%) than individuals who did not drop out.

### Attractiveness of contact- and education-based interventions

Table 1 shows the expected and observed median times for each intervention. The median times for the CC, CV, and EV groups were just above the expected intervention times. By contrast, for the other groups, median times were mostly far below the expected times. The biggest difference was found in the EC group, with an expected time of 170.5 s, but an observed median time of 79 s.

The median intervention exposure scores in the CC and CV groups were significantly higher than the median intervention exposure score in the CT group. Similarly, within the education groups, the median intervention exposure score for the EV group was significantly higher than the median intervention exposure scores for the EC and ET groups. Furthermore, the median intervention exposure scores for the CC and CT groups were significantly higher than the median intervention exposure scores in the same education groups. We found no such time differences between the CV and the EV group.

**Table 3:** Results of t-tests and χ^2^-tests to examine differences between individuals with different intervention times, *N* = 2,043

Variable	Unrealistically short intervention time (*n* = 595)	Within expected intervention time (*n* = 1,448)	t-value/ χ^2^	df	*p*
Age, *M* (*SD*)	51.40 (15.25)	48.54 (15.54)	3.83	1125.1	< 0.001
Gender, *n* (%)			12.27	2	0.002
Female Male Diverse	257 (43.2%) 337 (56.6%) 1 (0.2%)	747 (51.6%) 697 (48.1%) 4 (0.3%)			
Current Suicidal Thoughts, *n* (%)			0.00	1	1.00
No Yes	485 (81.5%) 110 (18.5%)	1180 (81.5%) 268 (18.5%)			
Lifetime Suicidality, *n* (%)			7.12	1	0.008
No Yes	446 (75.0%) 149 (25.0%)	998 (68.9%) 450 (31.1%)			
Education, *n* (%)			5.82	2	0.055
Low Middle High	231 (38.8%) 202 (33.9%) 162 (27.2%)	531 (36.7%) 445 (30.7%) 472 (32.6%)			
Suicide stigma t_0_, *M* (*SD*)	2.10 (0.85)	1.94 (0.78)	3.76	1023.5	< 0.001
Suicide normalization t_0_, *M* (*SD*)	2.13 (1.04)	2.11 (1.00)	0.37	1070.3	0.715

To inspect whether participants with an unrealistically short intervention time differ from other participants, we split the sample into two subsamples: participants with an intervention time shorter than half of the expected intervention time (i.e. intervention exposure score < -0.50; *n* = 595) and participants with an intervention time equal or longer than half of the expected intervention time (*n* = 1,448). Table 3 illustrates differences between these two samples in terms of sociodemographic characteristics, experiences with suicide ideation, and outcome measures at t_0_. Participants with an unrealistically short intervention time were significantly older, were more likely male than expected, reported less lifetime suicidality, and had higher suicide stigma at t_0_.

### Efficacy of contact- and education-based interventions

Table 4 shows the results of the linear mixed models to predict suicide stigma and suicide normalization. We found hardly any significant Time x Group interactions, i.e. there were no significant differences between participants exposed to the interventions and those exposed to the control condition with regard to changes in suicide stigma and suicide normalization. There was one exception: Among participants exposed to the contact-based video intervention (CV), there was a significantly stronger decrease in suicide normalization (at *p* < .05, cohen’s *d* = -0.13) between t_0_ and t_2_ compared to participants in the control condition (CC).

**Table 4:** Results of linear mixed models predicting suicide stigma and suicide normalization, *N* = 2,043

Predictors	Contact intervention						Education intervention					
	Baseline to post-intervention ^b^			Baseline to follow-up ^b^			Baseline to post-intervention ^c^			Baseline to follow-up ^c^		
	*B*	*SE*	*p*	*B*	*SE*	*p*	*B*	*SE*	*p*	*B*	*SE*	*p*
	*Suicide Stigma*											
Intercept	2.00	0.04	< 0.001	2.00	0.04	< 0.001	1.97	0.04	< 0.001	1.97	0.04	< 0.001
Time	-0.17	0.03	< 0.001	-0.05	0.03	0.154	-0.20	0.03	< 0.001	-0.07	0.03	0.036
Text intervention ^a^	0.03	0.06	0.625	0.03	0.06	0.624	-0.05	0.06	0.425	-0.05	0.06	0.435
Video intervention ^a^	0.06	0.06	0.329	0.06	0.06	0.329	-0.03	0.06	0.561	-0.03	0.06	0.569
Time x Text intervention ^a^	-0.03	0.04	0.483	0.01	0.05	0.794	0.03	0.04	0.489	0.02	0.05	0.692
Time x Video intervention ^a^	-0.06	0.04	0.108	-0.04	0.05	0.425	-0.03	0.04	0.413	0.03	0.05	0.587
	*Suicide Normalization*											
Intercept	2.11	0.05	< 0.001	2.11	0.05	< 0.001	2.09	0.06	< 0.001	2.09	0.06	< 0.001
Time	-0.05	0.03	0.132	0.04	0.04	0.316	-0.09	0.03	0.008	-0.00	0.04	0.945
Text intervention ^a^	0.01	0.08	0.928	0.01	0.08	0.926	-0.01	0.08	0.935	-0.01	0.08	0.934
Video intervention ^a^	0.04	0.08	0.618	0.04	0.08	0.610	0.06	0.08	0.403	0.06	0.08	0.398
Time x Text intervention ^a^	-0.02	0.05	0.606	-0.00	0.05	0.939	-0.08	0.05	0.105	-0.06	0.05	0.275
Time x Video intervention ^a^	-0.03	0.04	0.530	-0.13	0.05	0.016	-0.06	0.05	0.209	-0.04	0.05	0.454

^a^Reference group = Control condition

^b^Number of observations: 2042. Number of participants: 1021

^c^Number of observations: 2044. Number of participants: 1022

**Fig. 2:**
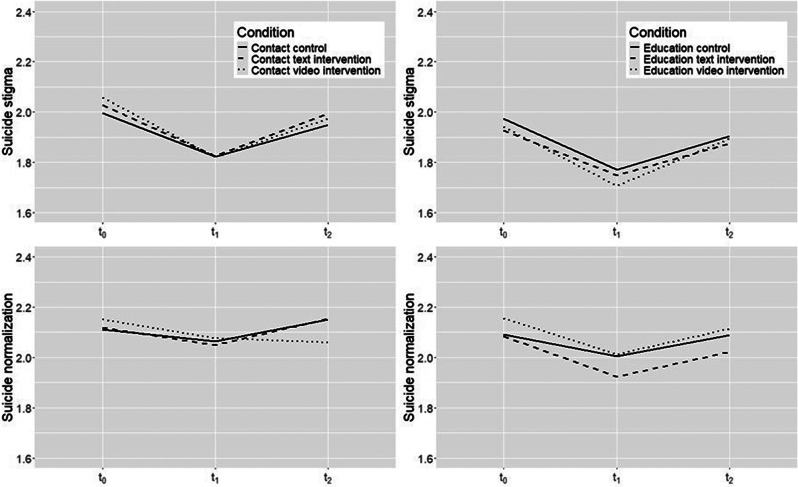
Changes in suicide stigma and suicide normalization across intervention groups

Figure 2 shows changes in suicide stigma and suicide normalization from baseline assessment (t_0_) via post-intervention assessment (t_1_) to follow-up assessment (t_2_) across groups. Interestingly, both average suicide normalization and particularly suicide stigma appear to decrease from t_0_ to t_1_ among participants exposed to the video and text interventions, including the two control groups. The lack of significant effects in the linear mixed models is therefore likely due to the observed decreases in suicide stigma and suicide normalization among participants in the control groups. This notion is supported by the results of the linear mixed models, which show (see Table 3, coefficient Time) a significant decrease in suicide stigma (contact- and education-based groups) and suicide normalization (education-based groups) in the CC and EC groups from t_0_ to t_1_. At t_2_, average suicide stigma and suicide normalization generally appear to return to their baseline levels across all intervention groups.

### Sensitivity analysis

We repeated the linear mixed models with a subsample of participants who met stringent quality criteria (*n* = 1,150) and with a subsample that included participants who dropped out between t_1_ and t_2_ (*n* = 3,702; intention-to-treat analysis). The smaller subsample of 1,150 individuals excluded participants with (a) inattentive responses indicated by incorrect replies on quality check items, (b) unrealistic (speedy) response times in filling in provided questionnaires using the 10th percentile for both waves, and (c) unrealistic intervention times (intervention exposure score < -0.50). In both subsamples, the pattern of results was very similar compared to the results with the sample used for main analyses (see online supplements A and B).

## Discussion

This study set out to examine the efficacy of contact and education-based interventions on suicide stigma and suicide normalization among members of the general population, as well as investigate the attractiveness of these interventions as indicated by the time participants spent with the provided intervention material. Below we discuss our findings in light of previous studies and make suggestions for future research and suicide prevention practice.

As indicated by calculated intervention exposure times, we observed higher attractiveness of video-based interventions compared to text-based interventions, as well as of contact material compared to education material, if transferred via text. Notably, those with high suicide stigma scores at baseline spent less time with provided intervention material and were more likely to drop-out, suggesting the need to identify strategies to attract the attention of those with increased intervention needs. As we investigated intervention effects among a general population sample with low suicide risk, provided intervention material may have little personal relevance for most participants. The low attractiveness of some of the tested interventions and the drop-out of participants with high suicide stigma scores likely contributed to the observed absence of efficacy (see below).

Surprisingly, compared to control conditions, none of the implemented interventions led to significant changes in suicide stigma or suicide normalization. The development of the tested interventions was based on a plethora of research providing evidence for effective stigma reduction based on contact- and education-based approaches [7, 18, 34]. For example, Winkler & colleagues [35] conducted a multi-center RCT to assess whether a brief (about 7 min) video-based contact intervention could reduce mental illness stigma among nursing students. They observed a significant medium sized reduction in mental illness stigma among those who watched the video, which was sustained at 3 months follow-up. Our non-significant findings and those of previous research [20, 21] suggest that public attitudes about suicide might be more persistent and less susceptible to change. This could potentially be explained by differences in the underlying beliefs and values that shape stigmatizing attitudes towards different social groups. Unlike attitudes about mental illness, attitudes about suicide might to a great extent depend on deeply personal values about life and death, personal liberty and religious beliefs, potentially making attitudinal change more difficult to achieve. Future research should investigate whether changes in intervention intensity (e.g. duration, repetition), intervention type (e.g. video, in-person) and intervention content (e.g. myths busting, narration of a personal recovery story) can lead to changes in suicide stigma.

Notably, despite non-significant Time x Group interactions, both suicide stigma and suicide normalization decreased from baseline to post-intervention across all groups, including controls. While this is surprising, as we used self-report questionnaires and informed participants about the aim of the study, observed reductions may be explained by demand characteristics, a common response bias in experimental research [36]. Its occurrence is generally based on participants’ desire to meet the expectations of the researcher, resulting in distorted response behavior. Participants were informed about the aim of the study at baseline and it is therefore likely that they developed assumptions about the research design. In line with that, as presented in the online supplements C and D, we observed significant improvements in suicide literacy for both video- and text-based interventions as compared to controls. Contrary to suicide stigma and suicide normalization, suicide literacy is knowledge-based and might therefore be less susceptible to biased response behavior. This suggests that participants did pay attention to the provided intervention material, while potential intervention effects were obscured by demand characteristics. To avoid bias due to demand characteristics, future research could implement implicit attitudinal measures rather than self-report questionnaires to measure suicide stigma, such as the brief implicit association test [37].

Our findings are subject to several limitations. Participants were recruited from a large pre-established online panel and while using quotas, the analyzed sample is not representative of the German general population. Some existing studies indicate sufficient validity of online-panel data [38], and our sensitivity analyses conducted among a subsample selected based on stringent quality criteria did also not indicate bias based on speedy or dishonest data entry. However, as outlined above, presumed demand characteristics could limit the validity of our findings. The exclusion of persons who indicated high distress by *suicidal thoughts during the past three months* during the screening limits the representativeness of our sample. Interestingly, despite this exclusion, 18.5% of participants reported *current suicidal thoughts* during the baseline assessment. While informing interested persons about the aim of the study was necessary from an ethical point of view, it likely introduced selection bias. People with higher suicide stigma scores at baseline were more likely to drop-out, making reductions in suicide stigma more difficult to achieve (i.e. floor effects). Finally, for text-based content, we defined the expected intervention time as the median reading time of eight research assistants, what might not reflect reading speed among members of the general population [39].

## Conclusions

Our findings suggest that changes in public suicide stigma are difficult to achieve. Future research should use experimental designs to test whether interventions with higher intensity and attractiveness lead to changes in public suicide stigma and suicide normalization among members of the general population. For example, researchers could investigate the effects of personal contact (as compared to video-based contact used in this study) or use smartphone technology to repeatedly engage participants with intervention material. The development and implementation of implicit measures could potentially help to avoid bias due to demand characteristics. Once identified, effective intervention components could be implemented within suicide prevention campaigns (SPCs), which aim for reduced suicide rates by changing relevant attitudes, beliefs and behaviors among members of the general population [40]. Despite great relevance for suicide prevention, the potential of using SPCs to reduce public suicide stigma remains unclear.

## Electronic supplementary material

Below is the link to the electronic supplementary material.

Supplementary Material 1

## Data Availability

Data supporting study findings are available from the corresponding author upon reasonable request.

## Abbreviations

CC: Contact-based control content
CT: Contact-based text intervention
CV: Contact-based video intervention
EC: Education-based control content
ET: Education-based text intervention
EV: Education-based video intervention
PHQ-9: Patient Health Questionnaire-9
RCT: Randomized controlled trial
SPCs: Suicide prevention campaigns
