# Supplementary material for: A web-based pilot randomized controlled trial to test the efficacy of education and contact-based interventions in reducing public suicide stigma
Source: BMC Psychiatry. 2025 Jan 23;25:70. doi: 10.1186/s12888-024-06406-7 (PMC11759414; doi:10.1186/s12888-024-06406-7)
Supplement: Supplementary file 1 — Supplementary Material 1 [file 12888_2024_6406_MOESM1_ESM.docx]

**Online Supplement A***: Results of linear mixed models with a subsample who met stringent quality criteria, N = 1,150*

| Predictors | Contact intervention | | | | | | Education intervention | | | | | |
| --- | --- | --- | --- | --- | --- | --- | --- | --- | --- | --- | --- | --- |
|  | Baseline to  post-intervention ^b^ | | | Baseline to  follow-up ^b^ | | | Baseline to  post-intervention ^c^ | | | Baseline to  follow-up ^c^ | | |
|  | *B* | *SE* | *p* | *B* | *SE* | *p* | *B* | *SE* | *p* | *B* | *SE* | *p* |
|  | *Suicide Stigma* | | | | | | | | | | | |
| Intercept | 1.95 | 0.05 | < .001 | 1.95 | 0.05 | < .001 | 1.96 | 0.07 | < .001 | 1.96 | 0.07 | < .001 |
| Time | -0.20 | 0.03 | < .001 | -0.08 | 0.04 | .032 | -0.24 | 0.04 | < .001 | -0.11 | 0.05 | .025 |
| Text intervention ^a^ | -0.06 | 0.08 | .465 | -0.06 | 0.08 | .463 | -0.19 | 0.10 | .048 | -0.19 | 0.10 | .051 |
| Video intervention ^a^ | 0.07 | 0.07 | .313 | 0.07 | 0.07 | .310 | -0.06 | 0.08 | .449 | -0.06 | 0.08 | .456 |
| Time x Text intervention ^a^ | 0.06 | 0.04 | .162 | 0.07 | 0.05 | .218 | 0.05 | 0.06 | .375 | 0.08 | 0.07 | .243 |
| Time x Video intervention ^a^ | -0.02 | 0.04 | .589 | 0.01 | 0.05 | .801 | 0.00 | 0.05 | .969 | 0.05 | 0.06 | .438 |
|  | *Suicide Normalization* | | | | | | | | | | | |
| Intercept | 2.11 | 0.07 | < .001 | 2.11 | 0.06 | < .001 | 1.96 | 0.09 | < .001 | 1.96 | 0.09 | < .001 |
| Time | -0.06 | 0.04 | .098 | 0.01 | 0.05 | .897 | -0.10 | 0.05 | .049 | 0.00 | 0.06 | .971 |
| Text intervention ^a^ | 0.01 | 0.10 | .944 | 0.01 | 0.10 | .943 | 0.08 | 0.13 | .534 | 0.08 | 0.13 | .538 |
| Video intervention ^a^ | -0.04 | 0.09 | .698 | -0.04 | 0.09 | .690 | 0.20 | 0.11 | .079 | 0.20 | 0.11 | .082 |
| Time x Text intervention ^a^ | -0.05 | 0.06 | .398 | -0.03 | 0.07 | .628 | -0.14 | 0.07 | .047 | -0.10 | 0.08 | .212 |
| Time x Video intervention ^a^ | -0.04 | 0.05 | .486 | -0.10 | 0.06 | .111 | -0.06 | 0.06 | .292 | -0.07 | 0.07 | .289 |

^a^ Reference group = Control condition.

^b^ Number of observations: 1304. Number of participants: 652.

^c^ Number of observations: 996. Number of participants: 498.

**Online Supplement B***: Results of linear mixed models with a subsample that includes participants who dropped out, N = 3,702*

| Predictors | Contact intervention | | | | | | Education intervention | | | | | |
| --- | --- | --- | --- | --- | --- | --- | --- | --- | --- | --- | --- | --- |
|  | Baseline to  post-intervention ^b^ | | | Baseline to  follow-up ^b^ | | | Baseline to  post-intervention ^c^ | | | Baseline to  follow-up ^c^ | | |
|  | *B* | *SE* | *p* | *B* | *SE* | *p* | *B* | *SE* | *p* | *B* | *SE* | *p* |
|  | *Suicide Stigma* | | | | | | | | | | | |
| Intercept | 2.07 | 0.03 | < .001 | 2.07 | 0.03 | < .001 | 2.05 | 0.03 | < .001 | 2.05 | 0.03 | < .001 |
| Time | -0.17 | 0.02 | < .001 | -0.07 | 0.03 | .036 | -0.19 | 0.02 | < .001 | -0.09 | 0.03 | .006 |
| Text intervention ^a^ | 0.01 | 0.05 | .849 | 0.01 | 0.05 | .848 | -0.01 | 0.05 | .837 | -0.01 | 0.05 | .835 |
| Video intervention ^a^ | 0.04 | 0.05 | .462 | 0.04 | 0.05 | .456 | 0.01 | 0.05 | .795 | 0.01 | 0.05 | .793 |
| Time x Text intervention ^a^ | -0.03 | 0.03 | .364 | 0.01 | 0.05 | .774 | 0.05 | 0.03 | .067 | 0.00 | 0.05 | .921 |
| Time x Video intervention ^a^ | -0.02 | 0.03 | .453 | -0.01 | 0.05 | .878 | -0.04 | 0.03 | .137 | 0.01 | 0.04 | .853 |
|  | *Suicide Normalization* | | | | | | | | | | | |
| Intercept | 2.14 | 0.04 | < .001 | 2.14 | 0.04 | < .001 | 2.08 | 0.04 | < .001 | 2.08 | 0.04 | < .001 |
| Time | -0.05 | 0.02 | .031 | 0.04 | 0.04 | .321 | -0.07 | 0.02 | .005 | 0.00 | 0.04 | .990 |
| Text intervention ^a^ | -0.03 | 0.06 | .600 | -0.03 | 0.05 | .597 | -0.01 | 0.06 | .834 | -0.01 | 0.06 | .834 |
| Video intervention ^a^ | 0.04 | 0.06 | .450 | 0.04 | 0.06 | .447 | 0.07 | 0.06 | .209 | 0.07 | 0.06 | -208 |
| Time x Text intervention ^a^ | -0.04 | 0.03 | .196 | 0.00 | 0.05 | .985 | -0.06 | 0.03 | .068 | -0.06 | 0.05 | .264 |
| Time x Video intervention ^a^ | -0.03 | 0.03 | .447 | -0.13 | 0.05 | .012 | -0.06 | 0.03 | .079 | -0.04 | 0.05 | .394 |

^a^ Reference group = Control condition.

^b^ Number of observations: 2873. Number of participants: 1844.

^c^ Number of observations: 2883. Number of participants: 1858.

**Online Supplement C:**

We found no support for significantly stronger effects among participants in text and video groups than among participants in control groups, which could potentially be explained by biased response behavior. We therefore used suicide literacy (i.e. factual knowledge about suicide and suicide prevention) as a more robust outcome measure that may be less susceptible to response bias. Suicide literacy was measured by the German version of the Literacy of Suicide Scale Short Form (LOSS-SF) (Calear et al., 2022), including 12 statements about suicide (e.g. “Seeing a psychiatrist or psychologist can help prevent someone from suicide.”) that are either right or wrong. Suicide literacy represents the sum of correctly classified statements as true or false (0-12). Rerunning the linear mixed models with suicide literacy as an outcome, we observed a significantly stronger increase in suicide literacy from t_0_ to t_1_ among participants who were exposed to the text (contact: *B* = 0.39, *SE* = 0.11, *p* < .001, cohen’s *d* = 0.17; education: *B* = 0.86, *SE* = 0.13, *p* < .001, cohen’s *d* = 0.36) and video interventions (contact: *B* = 0.31, *SE* = 0.10, *p* = .002, cohen’s *d* = 0.14; education: *B* = 1.38, *SE* = 0.12, *p* < .001, cohen’s *d* = 0.57) compared to participants in the control groups. Participants in the ET group (*B* = 0.60, *SE* = 0.15, *p* < .001, cohen’s *d* = 0.25) and the EV group (*B* = 0.40, *SE* = 0.14, *p* = .005, cohen’s *d* = 0.17) also showed a significantly stronger increase in suicide literacy between t_0_ and t_2_ compared to participants in the EC group. For a visual presentation of results, see Online Supplement D.

Calear, A. L., Batterham, P. J., Trias, A. and Christensen, H. (2022) 'The Literacy of Suicide Scale', *Crisis*, vol. 43, no. 5, pp. 385–390.

**Online Supplement D**: *Changes in Suicide Literacy across Intervention Groups*

*
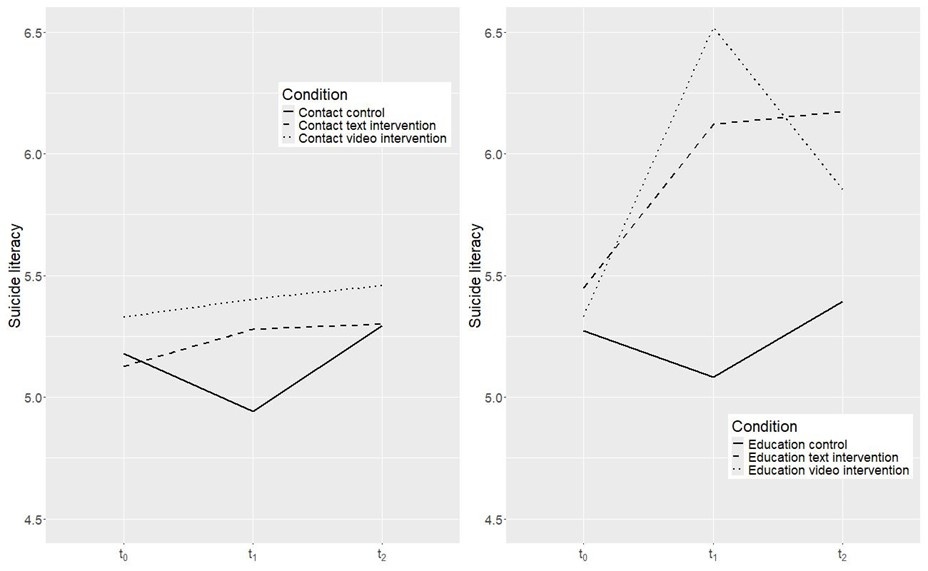
*
